# Hall Effect on Radiative Casson Fluid Flow with Chemical Reaction on a Rotating Cone through Entropy Optimization

**DOI:** 10.3390/e22040480

**Published:** 2020-04-22

**Authors:** Wejdan Deebani, Asifa Tassaddiq, Zahir Shah, Abdullah Dawar, Farhad Ali

**Affiliations:** 1 Department of Mathematics, College of Science and Arts, Rabigh King Abdul-Aziz University, Rabigh 21911, Saudi Arabia; wdeebani@kau.edu.sa; 2 College of Computer and Information Sciences Majmaah University, Al Majmaah 11952, Saudi Arabia; 3 Center of Excellence in Theoretical and Computational Science (TaCS-CoE), SCL 802 Fixed Point Laboratory, Science Laboratory Building, King Mongkut’s University of Technology Thonburi (KMUTT), Bangkok 10140, Thailand; 4 Department of Mathematics, Abdul Wali Khan University, Mardan 23200, Pakhtunkhwa, Pakistan; abdullah.mathematician@gmail.com; 5 Department of Mathematics, City University of Science and Information Technology, Peshawar 25000, KPK, Pakistan; farhadali@cusit.edu.pk

**Keywords:** entropy, thermal radiation, casson fluid, hall effect, chemical reaction, rotating cone

## Abstract

Magnetohydrodynamic (MHD) flow with Hall current has numerous applications in industrial areas such as Hall current accelerators, MHD power generators, planetary dynamics, Hall current sensors, etc. In this paper, the analysis of an unsteady MHD Casson fluid with chemical reaction over a rotating cone is presented. The impacts of Hall current, joule heating, thermal radiation, and viscous dissipation are analyzed. Entropy optimization is also considered in the present analysis. The system of coupled equations is tackled with homotopy analysis method (HAM). The convergence of HAM is also shown through figures. Deviations in the flow due to dimensionless parameters are shown graphically. Similarly, the variation in skin friction, Nusselt number, and Sherwood number are deliberated through tables. A justification of the current consequences is presented.

## 1. Introduction

The fluid flows through a cone have inspired attention due to recent improvements of innovative technologies. Fluid flows have excellent applications in many engineering and industrial fields like aeronautical engineering, solar collectors, rotating heat exchangers, homeotherapy treatment, endoscopy scanning, electronic chips, etc. Keeping in mind the significance of rotating flows, Kumari et al. [1] first presented the convective flow through a vertical cone. Nadeem and Saleem [2] analyzed the unsteady convective magnetohydrodynamic (MHD) flow over a rotating cone. Hayat et al. [3] explored the irreversibility characterization of the convective fluid flow through a rotating cone. The MHD radiative flow with Soret and chemical reaction through a rotating cone was numerically analyzed by Sulochana et al. [4]. Raju and Sandeep [5] investigated the MHD bioconvective flow through a rotating cone. Zohraa et al. [6] probed the bioconvective nanofluid flow over a rotating cone. Raju and Sandeep [7] numerically analyzed the flow of a Casson fluid through a rotating cone. Nadeem and Saleem [8] analyzed the nanofluid through a rotating cone. The convective fluid flow with suction/injection over a rotating cone was presented by Ravindran et al. [9]. Chamkha and Al Mudhaf [10] investigated the mass and heat transmission in MHD fluid flow. 

The analysis of MHD flow with Hall currents has numerous applications in industrial areas such as Hall currents accelerators, MHD power generators, planetary dynamics, Hall current sensors, etc. Initially, Sato [11] investigated the Hall current influence on the ionized gas flow in parallel plates. Sherman and Sutton [12] presented the Hall current impact on MHD generator efficiency. Katagiri [13] examined the MHD boundary layer flow with Hall currents. Pop and Soundalgekar [14] determined the hydrodynamic flow with Hall current influence. Gupta [15] and Jana et al. [16] probed the Hall current effect on hydrodynamic flow over a plate. Pop and Watanabe [17] studied the MHD boundary layer flow with Hall current. Aziz [18] examined the thermal transmission in a fluid flow with Hall Effects. Seth and Singh [19] investigated the convective hydrodynamic flow with Hall current. Wahed [20] presented the MHD nanofluid flow with radiation and Hall current. Osalusi et al. [21] offered the unsteady MHD mixed convective flow with variable properties through a rotating cone.

Different fluids, such as viscoelastic fluid, Williamson fluid, Jeffrey fluid, micropolar fluid, power-law fluid, Casson fluid, etc., are named as non-Newtonian fluids. A model of the Casson fluid [22] is presented in 1959. The MHD Casson fluid flow under the influences of Dufour and Soret impacts was investigated by Hayat et al. [23]. Eldabe and Salwa [24] deliberated the MHD flow of Casson fluid in a rotating cylinder. The Casson fluid flow over an extending surface was determined by Malik et al. [25]. Aziz and Afify [26] presented the thermal transfer in MHD radiative Casson fluid. Shateyi et al. [27] observed the convective thermal and mass transmission in a Casson fluid flow with viscous dissipation effect. The Casson fluid flow with magnetic influence was determined by Shehzad et al. [28]. Reddy et al. [29] analyzed the thermal transmission in a Casson fluid flow over a thermal sheet. The above studies were based on the first law of thermodynamics. 

It is well known that entropy, as a thermodynamic function, reflects a system’s operating status. At the same time, it is necessary to minimize the entropy generation of a system to improve its working effectiveness. Entropy generation minimization techniques can be employed for the optimization of technical systems including heat exchangers, elements of nuclear and thermal power plants, ventilation and air conditioning systems, and so on. Thermodynamic second laws are utilized to examine the entropy optimization in term of the entropy age rate. Entropy augmentation is exploited to elucidate the performance of dissimilar contexts in modern and structure applications. Entropy is imitative from Greek word entropia, which implies that “a moving in the direction of” or “change”. Entropy calculation is essential as it classifies the parameters for energy loss. Alternatively, thermodynamics second law was employed to minimalize the entropy optimization in engineering systems by Bejan [30,31]. Using different geometrical configurations, the authors of [32,33,34,35,36] have calculated the entropy generation. Chen et al. [37] and Liu and Lo [38] analyzed the entropy optimization in convective flow with viscous dissipation influence. Shah et al. [39,40] recently investigated entropy optimization in a nanofluid flow in different geometries. Numerical and analytical approaches are used in their work for results. The others related investigations to MHD Casson fluid are mentioned in [39,40,41,42,43,44,45,46] 

This work presents the analysis of MHD Casson fluid flow with chemical reaction over a rotating cone. Joule heating, radiation, viscous impact, and Hall current are deliberated in this work. Furthermore, the features of entropy generation with first-order chemical reaction are examined. The system of coupled equations is tackled with HAM. The convergence of HAM is also shown in the following figures. Deviation in the flow distributions due to dimensionless factors are published through figures. The validation of the present results is presented through tables. Table 1 led us to continue our analysis on Casson fluid flow with chemical reaction and entropy generation. 

## 2. Problem Modeling 

**Figure 1:**
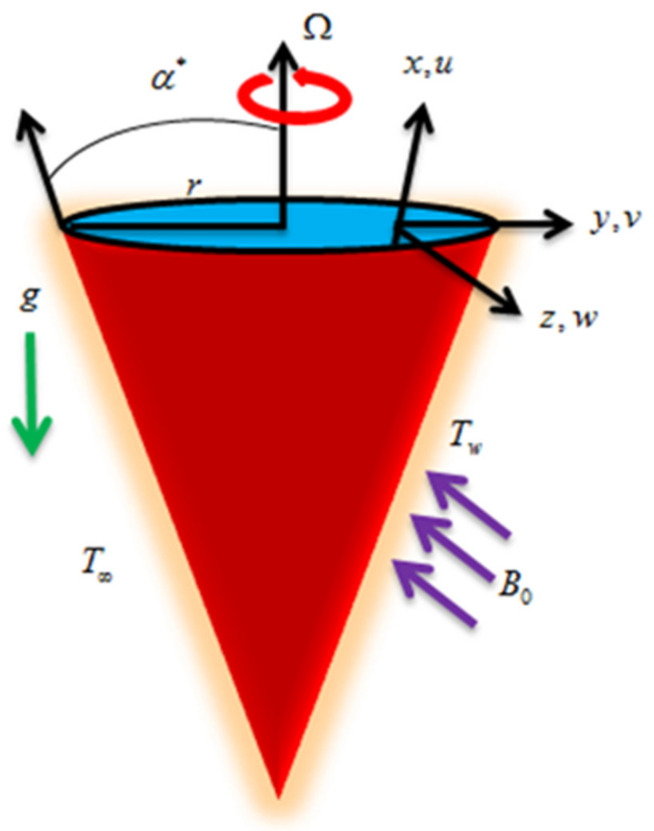
Fluid flow geometry.

Unsteady and incompressible Casson fluid flow with chemical reaction by a rotating cone is considered here. The cone rotates with angular velocity Ω as shown in Figure 1. The gravitational acceleration g acts downward. Thermal radiation, Joule heating, and viscous dissipation are demonstrated in the temperature equation. Furthermore, the entropy generation is also considered. 

The general Ohm’s law with Hall current is defined as

(1) $$\vec{J}+\frac{\omega_{e}\tau_{e}}{B_{0}}\left(\vec{J}\times \vec{B}\right)=\sigma \left(\vec{E}+\vec{V}\times \vec{B}\right),$$

Here, in this analysis, the electric field is ignored. Therefore, Equation (1) reduced as

(2) $$J_{x}=\frac{\sigma B_{0}}{\left(1+m^{2}\right)}\left(mu-v\right),$$

(3) $$J_{y}=\frac{\sigma B_{0}}{\left(1+m^{2}\right)}\left(mv+u\right),$$

in which $m=\tau_{e}\omega_{e}$ represents the Hall factor.

Furthermore, the basic model of the Casson fluid is demarcated as

(4) $$\tau_{ij}=\left\{\begin{array}{l} 2\left(\mu_{B}+\frac{S_{y}}{{\left(2\chi \right)}^{1/2}}\right)e_{ij},\;\;\chi >\chi_{c}\\ 2\left(\mu_{B}+\frac{S_{y}}{{\left(2\chi_{c}\right)}^{1/2}}\right)e_{ij},\;\;\chi <\chi_{c} \end{array}\right.$$

where $\chi =e_{ij}e_{ij}$ is the product of deformation rate components, $\chi_{c}$ represents the critical value of this product, $\mu_{B}$ is the plastic dynamic viscosity, and $S_{y}$ is the fluid yield stress. When $\chi <\chi_{c}$, Equation (4) is summarized as

(5) $$\tau_{ij}=2e_{ij}\left(1+\frac{1}{\psi }\right)\mu_{B},$$

where $\psi =\frac{{\left(2\chi_{c}\right)}^{1/2}\mu_{B}}{S_{y}}$ is the Casson factor.

In view of the mentioned assumptions, the leading equations of the Casson fluid and the general Ohm’s law, including Hall current effect, are specified as

(6) $$\frac{\partial \left(xu\right)}{\partial x}+\frac{\partial \left(xw\right)}{\partial z}=0,$$

(7) $$\begin{array}{l} \frac{\partial u}{\partial t}+u\frac{\partial u}{\partial x}-\frac{v^{2}}{x}+w\frac{\partial u}{\partial z}=\nu \left(1+\frac{1}{\psi }\right)\frac{\partial^{2}u}{\partial z^{2}}-\frac{\sigma B_{0}^{2}}{\rho \left(1+m^{2}\right)}\left(u+mv\right)\\ +\;g\beta_{t}\cos\alpha^{*}\left(T-T_{\infty }\right)+g\beta_{c}\cos\alpha^{*}\left(C-C_{\infty }\right), \end{array}$$

(8) $$\frac{\partial v}{\partial t}+u\frac{\partial v}{\partial x}+\frac{uv}{x}+w\frac{\partial v}{\partial z}=\nu \left(1+\frac{1}{\psi }\right)\frac{\partial^{2}v}{\partial z^{2}}+\frac{\sigma B_{0}^{2}}{\rho \left(1+m^{2}\right)}\left(mu-v\right),$$

(9) $$\begin{array}{l} \frac{\partial T}{\partial t}+u\frac{\partial T}{\partial x}+w\frac{\partial T}{\partial z}=\frac{k}{\rho c_{p}}\frac{\partial^{2}T}{\partial z^{2}}+\frac{\mu }{\rho c_{p}}\left(1+\frac{1}{\psi }\right)\left[{\left(\frac{\partial u}{\partial z}\right)}^{2}+{\left(\frac{\partial v}{\partial z}\right)}^{2}\right]+\\ \frac{16}{3}\frac{\sigma^{*}T_{\infty }^{3}}{k^{*}\rho c_{p}}+\frac{\sigma B_{0}^{2}}{\rho c_{p}}\left(u^{2}+v^{2}\right), \end{array}$$

(10) $$\frac{\partial C}{\partial t}+u\frac{\partial C}{\partial x}+w\frac{\partial C}{\partial z}=D_{B}\frac{\partial^{2}C}{\partial z^{2}}-K_{r}\left(C-C_{\infty }\right),$$

with

(11) $$\begin{array}{l} \left.\begin{array}{l} u\left(x,\zeta ,t\right)=N_{0}\mu \frac{\partial u}{\partial z},\;\;v\left(x,\zeta ,t\right)=\frac{\Omega x\sin\alpha^{*}}{\left(1-qt\right)}+N_{0}\mu \frac{\partial v}{\partial z},\;\\ \;w\left(x,\zeta ,t\right)=0,\;\;T\left(x,\zeta ,t\right)=T_{w},\;\;C\left(x,\zeta ,t\right)=C_{w} \end{array}\right\}\;\;\mathrm{at}\;\;\zeta =0,\\ \left.u\left(x,\zeta ,t\right)=0,\;\;v\left(x,\zeta ,t\right)=0,\;\;T\left(x,\zeta ,t\right)=T_{\infty },\;\;C\left(x,\zeta ,t\right)=C_{\infty }\right\}\;\;\;\mathrm{at}\;\;\zeta =\infty .\;\; \end{array}$$

Here x,  y  and  z; u,  v  and  w; and μ, ρ,$\alpha^{*}$, $\beta_{t}$, $\beta_{c}$, $N_{0}$, $T,\;\;T_{w}$, $T_{\infty }$, $\sigma^{*}$, $c_{p}$, C, $C_{w}$, $C_{\infty }$, $D_{B}$, Ω, and $K_{r}$ are the coordinate axes, respectively; velocity components, correspondingly; dynamic viscosity, density, semi-vertical angle, thermal coefficient, concentration coefficient, velocity slip factor, temperature, temperature at the surface, temperature away from the surface, Stefan–Boltzman constant, specific heat, concentration, concentration at the surface, concentration away from the surface, mass diffusivity, dimensionless angular velocity, and chemical reaction rate, respectively.

Considering

(12) $$\begin{array}{l} u=-\frac{1}{2}\frac{\Omega x\sin\alpha^{*}}{\left(1-qt^{*}\right)}f^{\prime }\left(\zeta \right),\;\;\;v=\frac{\Omega x\sin\alpha^{*}}{\left(1-qt^{*}\right)}g\left(\zeta \right),\;\;\;w=\sqrt{\frac{\nu_{0}\Omega \sin\alpha^{*}}{\left(1-qt^{*}\right)}}f\left(\zeta \right),\;\\ \;\theta \left(\zeta \right)=\frac{T-T_{\infty }}{T_{w}-T_{\infty }},\;\;\;\left(T_{w}-T_{\infty }\right)=\frac{\left(T_{0}-T_{\infty }\right)}{{\left(1-qt^{*}\right)}^{2}}\frac{x}{L},\;\;\;\phi \left(\zeta \right)=\frac{C-C_{\infty }}{C_{w}-C_{\infty }},\;\;\\ \left(C_{w}-C_{\infty }\right)=\frac{\left(C_{0}-C_{\infty }\right)}{{\left(1-qt^{*}\right)}^{2}}\frac{x}{L},\;\;\;\;t^{*}=\left(\Omega \sin\alpha^{*}\right)t,\;\;\;\;\zeta =\sqrt{\frac{\Omega \sin\alpha^{*}}{\left(1-qt^{*}\right)\nu_{0}}}\;z. \end{array}$$

The transformed equations are

(13) $$\begin{array}{l} \left(1+\frac{1}{\psi }\right)f^{\prime \prime \prime }\left(\zeta \right)+\frac{1}{2}{f^{\prime }}^{2}\left(\zeta \right)-2g^{2}\left(\zeta \right)-f\left(\zeta \right)f^{\prime \prime }\left(\zeta \right)-S\left(f^{\prime }\left(\zeta \right)+\frac{\zeta }{2}f^{\prime \prime }\left(\zeta \right)\right)\\ -2\lambda \left(\theta \left(\zeta \right)+N\phi \left(\zeta \right)\right)-\frac{M}{1+m^{2}}\left(f^{\prime }\left(\zeta \right)+mg\left(\zeta \right)\right)=0, \end{array}$$

(14) $$\begin{array}{l} \left(1+\frac{1}{\psi }\right)g^{\prime \prime }\left(\zeta \right)+g\left(\zeta \right)f^{\prime }\left(\zeta \right)-f\left(\zeta \right)g^{\prime }\left(\zeta \right)-\\ S\left(g\left(\zeta \right)+\frac{\zeta }{2}g^{\prime }\left(\zeta \right)\right)+\frac{M}{1+m^{2}}\left(mf^{\prime }\left(\zeta \right)-g\left(\zeta \right)\right)=0, \end{array}$$

(15) $$\begin{array}{l} \left(1+Rd\right)\theta^{\prime \prime }\left(\zeta \right)+\frac{1}{2}\mathrm{Pr}f^{\prime }\left(\zeta \right)\theta \left(\zeta \right)-\mathrm{Pr}f\left(\zeta \right)\theta^{\prime }\left(\zeta \right)-\mathrm{Pr}S\left(2\theta \left(\zeta \right)+\frac{\zeta }{2}\theta^{\prime }\left(\zeta \right)\right)\\ +Br\left(1+\frac{1}{\psi }\right)\left(\frac{1}{4}{f^{\prime \prime }}^{2}\left(\zeta \right)+{g^{\prime }}^{2}\left(\zeta \right)\right)+MBr\left(\frac{1}{4}{f^{\prime }}^{2}\left(\zeta \right)+g^{2}\left(\zeta \right)\right)=0, \end{array}$$

(16) $$\phi^{\prime \prime }\left(\zeta \right)+\frac{1}{2}Scf^{\prime }\left(\zeta \right)\phi \left(\zeta \right)-S\left(2\phi \left(\zeta \right)+\frac{\zeta }{2}\phi^{\prime }\left(\zeta \right)\right)-Scf\left(\zeta \right)\phi^{\prime }\left(\zeta \right)-\gamma Sc\phi \left(\zeta \right)=0,$$

(17) $$\begin{array}{l} f\left(\zeta \right)=0,\;\;f^{\prime }\left(\zeta \right)=\beta f^{\prime \prime }\left(\zeta \right),\;\;g\left(\zeta \right)=1+\beta g^{\prime }\left(\zeta \right),\;\;\theta \left(\zeta \right)=1,\;\;\phi \left(\zeta \right)=1\;\;\;\mathrm{at}\;\;\;\zeta =0,\\ f^{\prime }\left(\zeta \right)\to 0,\;\;g\left(\zeta \right)\to 0,\;\;\theta \left(\zeta \right)\to 0,\;\;\phi \left(\zeta \right)\to 0\;\;\;\mathrm{as}\;\;\zeta \to \infty . \end{array}$$

where Re, Gr, Pr, Ec, Br, and Sc denote the Reynolds, Grashof, Prandtl, Eckert, Brinkman, and Schmidt numbers, respectively. M, λ, N, β, Rd, and γ are magnetic, mixed convection, buoyancy ratio, velocity slip, radiation, and chemical reaction parameters, respectively, which are defined as

(18) Re=L2Ωsinα*ν,  Gr=gβtcosα*T0−T∞L3ν2,  M=σB02ρΩsinα*,Sc=νD,N=βcC0−C∞βtT0−T∞, λ=GrRe2,   Br=PrEc,  γ=krΩsinα*, β=N0Ωsinα*ν,  Pr=ν0α,  Ec=Ω2Lxsin2α*cpT0−T∞,  Rd=163σ*T∞3kk*.

### Velocity, Temperature, and Mass Gradients

The velocity ($C_{fx},\;\;C_{fy}$), temperature ($Nu_{x}$), and mass ($Sh_{x}$) gradients are

(19) $$C_{fx}=\frac{{\left.2\tau_{xz}\right|}_{z=0}}{\rho {\left(\frac{\Omega x\sin\alpha^{*}}{1-st^{*}}\right)}^{2}},\;\;\;C_{fy}=\frac{{\left.2\tau_{yz}\right|}_{z=0}}{\rho {\left(\frac{\Omega x\sin\alpha^{*}}{1-st^{*}}\right)}^{2}},\;\;Nu_{x}=\frac{{\left.xq_{w}\right|}_{z=0}}{k\left(T_{w}-T_{\infty }\right)},\;\;Sh_{x}=\frac{{\left.xh_{w}\right|}_{z=0}}{k\left(C_{w}-C_{\infty }\right)},$$

where $\tau_{xz}$ and $\tau_{yz}$ are shear stresses, $q_{w}$ and $h_{w}$ are heat and mass fluxes, which are defined as

(20) $$\begin{array}{l} \tau_{xz}=-\left(\mu_{B}+\frac{S_{y}}{{\left(2\chi_{c}\right)}^{1/2}}\right)\left(\frac{\partial u}{\partial z}\right),\;\;\tau_{yz}=-\left(\mu_{B}+\frac{S_{y}}{{\left(2\chi_{c}\right)}^{1/2}}\right)\left(\frac{\partial v}{\partial z}\right),\;\\ q_{w}=-\left(\frac{\partial T}{\partial z}\right)-\frac{16}{3}\frac{\sigma^{*}T_{\infty }^{3}}{k^{*}}\left(\frac{\partial T}{\partial z}\right),\;\;h_{w}=-D\left(\frac{\partial C}{\partial z}\right). \end{array}$$

The dimensionless forms are

(21) CfxRex1/2=−1+1ψf″0,     12CfyRex1/2=−1+1ψg′0,  NuxRex1/2=−1+Rdθ′0,    ShxRex1/2=−ϕ′0.

## 3. Entropy Optimization

Mathematical expression of entropy optimization is

(22) $$\begin{array}{l} S_{G}=\frac{k}{T_{\infty }^{2}}\left(1+\frac{16}{3}\frac{\sigma^{*}T_{\infty }^{3}}{k^{*}k}\right){\left(\frac{\partial T}{\partial z}\right)}^{2}+\frac{\mu_{B}}{T_{\infty }}\left(1+\frac{1}{\psi }\right)\left[{\left(\frac{\partial u}{\partial z}\right)}^{2}+{\left(\frac{\partial v}{\partial z}\right)}^{2}\right]\\ \;\;\;\;\;\;\;\;\;\;+\frac{R_{D}}{T_{\infty }}\left(\frac{\partial C}{\partial z}\frac{\partial T}{\partial z}\right)+\frac{R_{D}}{C_{\infty }}{\left(\frac{\partial C}{\partial z}\right)}^{2}+\frac{\mu_{B}}{T_{\infty }}\frac{\sigma B_{0}^{2}}{\rho \left(1+m^{2}\right)}\left(u^{2}+v^{2}\right), \end{array}$$

The dimensionless form is

(23) $$\begin{array}{l} N_{G}=T_{d}\left(1+Rd\right){\left(\theta^{\prime }\left(\zeta \right)\right)}^{2}+\frac{Br}{A}\left(1+\frac{1}{\psi }\right)\left(\frac{1}{4}{f^{\prime \prime }}^{2}\left(\zeta \right)+{g^{\prime }}^{2}\left(\zeta \right)\right)+\\ L\theta^{\prime }\left(\zeta \right)\phi^{\prime }\left(\zeta \right)+L\frac{C_{d}}{T_{d}}{\phi^{\prime }}^{2}\left(\zeta \right)+\frac{MBr}{A\left(1+m^{2}\right)}\left(\frac{1}{4}{f^{\prime }}^{2}\left(\zeta \right)+g^{2}\left(\zeta \right)\right), \end{array}$$

### Bejan Number

The Bejan number is defined as

(24) $$Be=\frac{\mathrm{Irreversibilities}\;\mathrm{due}\;\mathrm{to}\;\mathrm{heat}\;\mathrm{and}\;\mathrm{mass}\;\mathrm{transfer}}{\mathrm{Total}\;\mathrm{entropy}\;\mathrm{rate}},$$

(25) $$Be=\frac{T_{d}\left(1+Rd\right){\left(\theta^{\prime }\left(\zeta \right)\right)}^{2}+L\theta^{\prime }\left(\zeta \right)\phi^{\prime }\left(\zeta \right)+L\frac{C_{d}}{T_{d}}{\phi^{\prime }}^{2}\left(\zeta \right)}{\left[\begin{array}{l} \;\;\;T_{d}\left(1+Rd\right){\left(\theta^{\prime }\left(\zeta \right)\right)}^{2}+\frac{Br}{A}\left(1+\frac{1}{\psi }\right)\left(\frac{1}{4}{f^{\prime \prime }}^{2}\left(\zeta \right)+{g^{\prime }}^{2}\left(\zeta \right)\right)+\\ L\theta^{\prime }\left(\zeta \right)\phi^{\prime }\left(\zeta \right)+L\frac{C_{d}}{T_{d}}{\phi^{\prime }}^{2}\left(\zeta \right)+\frac{MBr}{A\left(1+m^{2}\right)}\left(\frac{1}{4}{f^{\prime }}^{2}\left(\zeta \right)+g^{2}\left(\zeta \right)\right) \end{array}\right]}.$$

where $N_{G}$ signifies the entropy rate; L indicates the diffusion parameter; $T_{d}$ and $C_{d}$ represent temperature difference and concentration difference, respectively; and A is the dimensionless parameter which are defined as

(26) $$N_{G}=\frac{\nu_{0}T_{\infty }S_{G}L^{2}}{k\left(T_{0}-T_{\infty }\right)\Omega x^{2}\sin\alpha^{*}},\;\;L=\frac{\left(C_{0}-C_{\infty }\right)R_{D}}{k},\;\;T_{d}=\frac{T_{0}-T_{\infty }}{T_{\infty }},\;\;C_{d}=\frac{C_{0}-C_{\infty }}{C_{\infty }},\;\;A=\frac{x}{L}.$$

## 4. HAM Solution 

The analytical solution of the modeled coupled equations given in Equations (13–16) is conceded by HAM. It is supposed that f=U,  g=V,  θ=T  and ϕ=C. The initial suppositions and linear operatives for velocities, temperature, and concentration functions, respectively, are given as

(27) $$U_{0}(\zeta )=0,\;\;V_{0}(\zeta )=\frac{1}{1+\beta }e^{-\zeta },\;\;T_{0}(\zeta )=e^{-\zeta },\;\;C_{0}(\zeta )=e^{-\zeta }.$$

(28) $$L_{U}\left(U\right)=U^{\prime \prime \prime }-U^{\prime },\;\;L_{V}\left(V\right)=V^{\prime \prime }-V,\;\;L_{T}\left(T\right)=T^{\prime \prime }-T,\;\;L_{C}\left(C\right)=C^{\prime \prime }-C.$$

With

(29) $$\begin{array}{l} L_{U}\left({\bar{Y}}_{1}+{\bar{Y}}_{2}e^{-\zeta }+{\bar{Y}}_{3}e^{\zeta }\right)=0,\;L_{V}\left({\bar{Y}}_{4}e^{-\zeta }+{\bar{Y}}_{5}e^{\zeta }\right)=0,\\ L_{T}\left({\bar{Y}}_{6}e^{-\zeta }+{\bar{Y}}_{7}e^{\zeta }\right)=0,\;\;\;L_{C}\left({\bar{Y}}_{8}e^{-\zeta }+{\bar{Y}}_{9}e^{\zeta }\right)=0. \end{array}$$

where ${\bar{Y}}_{1},{\bar{Y}}_{2},{\bar{Y}}_{3},\ldots ,{\bar{Y}}_{9}$ are constants.

$0^{th}-$ **order problems**

Let $Χ\in \left[0,\;\;1\right]$ and $\hbar_{U}$, $\hbar_{V}$, $\hbar_{T}$, $\hbar_{C}$ be the embedding and non-zero auxiliary factors, respectively, then

(30) $$\left(1-Χ\right)L_{U}\left[U\left(\zeta ;Χ\right)-U_{0}\left(Χ\right)\right]=Χ\hbar_{U}{ℵ}_{U}\left[U\left(\zeta ;Χ\right),V\left(\zeta ;Χ\right),T\left(\zeta ;Χ\right),C\left(\zeta ;Χ\right)\right],$$

(31) $$\left(1-Χ\right)L_{V}\left[V\left(\zeta ;Χ\right)-V_{0}\left(\zeta \right)\right]=Χ\hbar_{V}{ℵ}_{V}\left[V\left(\zeta ;Χ\right),U\left(\zeta ;Χ\right)\right],$$

(32) $$\left(1-Χ\right)L_{T}\left[T\left(\zeta ;Χ\right)-T_{0}\left(\zeta \right)\right]=Χ\hbar_{T}{ℵ}_{T}\left[T\left(\zeta ;Χ\right),U\left(\zeta ;Χ\right),V\left(\zeta ;Χ\right)\right],$$

(33) $$\left(1-Χ\right)L_{C}\left[C\left(\zeta ;Χ\right)-C_{0}\left(\zeta \right)\right]=Χ\hbar_{C}{ℵ}_{C}\left[C\left(\zeta ;Χ\right),U\left(\zeta ;Χ\right)\right],$$

(34) $$\begin{array}{l} \;\;\;\;\;\;\;\;\;\;\;U\left(0;Χ\right)=0,\;\;U^{\prime }\left(0;Χ\right)=\beta U^{\prime \prime }\left(0;Χ\right),\\ \;\;V\left(0;Χ\right)=1+\beta V^{\prime }\left(0;Χ\right),\;\;T\left(0;Χ\right)=C\left(0;Χ\right)=1\\ U^{\prime }\left(\infty ;Χ\right)=V\left(\infty ;Χ\right)=0,\;\;T\left(\infty ;Χ\right)=C\left(\infty ;Χ\right)=0, \end{array}$$

(35) $$\begin{array}{l} {ℵ}_{U}\left[U\left(\zeta ;Χ\right),V\left(\zeta ;Χ\right),T\left(\zeta ;Χ\right),C\left(\zeta ;Χ\right)\right]=\left(1+\frac{1}{\psi }\right)\frac{\partial^{3}U\left(\zeta ;Χ\right)}{\partial \zeta^{3}}+\frac{1}{2}{\left(\frac{\partial U\left(\zeta ;Χ\right)}{\partial \zeta }\right)}^{2}\\ \;\;\;\;\;\;\;\;\;-2{\left(V\left(\zeta ;Χ\right)\right)}^{2}-U\left(\zeta ;Χ\right)\frac{\partial^{2}U\left(\zeta ;Χ\right)}{\partial \zeta^{2}}-S\left(\frac{\partial U\left(\zeta ;Χ\right)}{\partial \zeta }+\frac{1}{2}\zeta \frac{\partial^{2}U\left(\zeta ;Χ\right)}{\partial \zeta^{2}}\right)\\ \;\;\;\;\;\;\;\;\;\;\;\;\;\;\;-2\lambda \left(T\left(\zeta ;Χ\right)+NC\left(\zeta ;Χ\right)\right)-\frac{M}{1+m^{2}}\left(\frac{\partial U\left(\zeta ;Χ\right)}{\partial \zeta }+mV\left(\zeta ;Χ\right)\right), \end{array}$$

(36) $$\begin{array}{l} {ℵ}_{V}\left[V\left(\zeta ;Χ\right),U\left(\zeta ;Χ\right)\right]=\left(1+\frac{1}{\psi }\right)\frac{\partial^{2}V\left(\zeta ;Χ\right)}{\partial \zeta^{2}}+V\left(\zeta ;Χ\right)\frac{\partial U\left(\zeta ;Χ\right)}{\partial \zeta }-U\left(\zeta ;Χ\right)\frac{\partial V\left(\zeta ;Χ\right)}{\partial \zeta }\\ \;\;\;\;\;\;\;\;\;\;\;\;\;\;\;\;\;\;-S\left(V\left(\zeta ;Χ\right)+\frac{1}{2}\zeta \frac{\partial V\left(\zeta ;Χ\right)}{\partial \zeta }\right)+\frac{M}{1+m^{2}}\left(m\frac{\partial U\left(\zeta ;Χ\right)}{\partial \zeta }-V\left(\zeta ;Χ\right)\right), \end{array}$$

(37) $$\begin{array}{l} \;\;\;\;\;\;\;\;\;{ℵ}_{T}\left[T\left(\zeta ;Χ\right),U\left(\zeta ;Χ\right),V\left(\zeta ;Χ\right)\right]=\left(1+Rd\right)\frac{\partial^{2}T\left(\zeta ;Χ\right)}{\partial \zeta^{2}}+\frac{1}{2}\mathrm{Pr}T\left(\zeta ;Χ\right)\frac{\partial U\left(\zeta ;Χ\right)}{\partial \zeta }\\ \;\;\;\;\;\;\;\;\;\;\;\;\;\;\;\;\;\;\;\;\;\;\;\;\;\;-\mathrm{Pr}U\left(\zeta ;Χ\right)\frac{\partial T\left(\zeta ;Χ\right)}{\partial \zeta }-S\mathrm{Pr}\left(2T\left(\zeta ;Χ\right)+\frac{\zeta }{2}\frac{\partial T\left(\zeta ;Χ\right)}{\partial \zeta }\right)+\\ Br\left(1+\frac{1}{\psi }\right)\left(\frac{1}{4}{\left(\frac{\partial^{2}U\left(\zeta ;Χ\right)}{\partial \zeta^{2}}\right)}^{2}+{\left(\frac{\partial V\left(\zeta ;Χ\right)}{\partial \zeta }\right)}^{2}\right)+MBr\left(\frac{1}{4}{\left(\frac{\partial U\left(\zeta ;Χ\right)}{\partial \zeta }\right)}^{2}+{\left(V\left(\zeta ;Χ\right)\right)}^{2}\right), \end{array}$$

(38) $$\begin{array}{l} \;\;\;\;\;\;\;\;{ℵ}_{C}\left[C\left(\zeta ;Χ\right);U\left(\zeta ;Χ\right)\right]=\frac{\partial^{2}C\left(\zeta ;Χ\right)}{\partial \zeta^{2}}+\frac{1}{2}ScC\left(\zeta ;Χ\right)\frac{\partial U\left(\zeta ;Χ\right)}{\partial \zeta }\\ -ScU\left(\zeta ;Χ\right)\frac{\partial C\left(\zeta ;Χ\right)}{\partial \zeta }-S\left(2C\left(\zeta ;Χ\right)+\frac{1}{2}\zeta \frac{\partial C\left(\zeta ;Χ\right)}{\partial \zeta }\right)-\gamma ScC\left(\zeta ;Χ\right). \end{array}$$

$t^{\mathrm{th}}-$ order problems are

(39) $$L_{U}\left[U_{t}\left(\zeta \right)-\chi_{t}U_{t-1}\left(\zeta \right)\right]=h_{t}{ℜ}_{t}^{U}\left(\zeta \right),$$

(40) $$L_{V}\left[V_{t}\left(\zeta \right)-\chi_{t}V_{t-1}\left(\zeta \right)\right]=h_{V}{ℜ}_{t}^{V}\left(\zeta \right),$$

(41) $$L_{T}\left[T_{t}\left(\zeta \right)-\chi_{t}T_{t-1}\left(\zeta \right)\right]=h_{T}{ℜ}_{t}^{T}\left(\zeta \right),$$

(42) $$L_{C}\left[C_{t}\left(\zeta \right)-\chi_{t}C_{t-1}\left(\zeta \right)\right]=h_{C}{ℜ}_{t}^{C}\left(\zeta \right),$$

(43) $$U_{t}\left(0\right)={U^{\prime }}_{t}\left(0\right)={U^{\prime }}_{t}\left(\infty \right)=0,\;\;\;\;\;V_{t}\left(0\right)=V_{t}\left(\infty \right)=0,\;\;\;\;T_{t}\left(0\right)=T_{t}\left(\infty \right)=0,\;\;\;\;C_{t}\left(0\right)=C_{t}\left(\infty \right)=0.$$

(44) $$\begin{array}{l} \;\;\;\;\;\;{ℜ}_{t}^{U}\left(\zeta \right)=\left(1+\frac{1}{\psi }\right){U^{\prime \prime \prime }}_{t-1}+\frac{1}{2}{U^{\prime }}_{t-1}^{2}-2V_{t-1}^{2}-\sum_{j=0}^{t-1}\left(U_{t-1-j}{U^{\prime \prime }}_{j}\right)-\\ S\left({U^{\prime }}_{t-1}+\frac{1}{2}\zeta {U^{\prime \prime }}_{t-1}\right)-2\lambda \left(T_{t-1}+NC_{t-1}\right)-\frac{M}{1+m^{2}}\left({U^{\prime }}_{t-1}+mV_{t-1}\right), \end{array}$$

(45) $$\begin{array}{l} {ℜ}_{t}^{V}\left(\zeta \right)=\left(1+\frac{1}{\psi }\right){V^{\prime \prime }}_{t-1}+\sum_{j=0}^{t-1}\left(V_{t-1-j}{U^{\prime }}_{j}\right)-\sum_{j=0}^{t-1}\left(U_{t-1-j}{V^{\prime }}_{j}\right)\\ \;\;\;\;\;\;\;\;\;\;\;\;-S\left(V_{t-1}+\frac{1}{2}\zeta {V^{\prime }}_{t-1}\right)+\frac{M}{1+m^{2}}\left(m{U^{\prime }}_{t-1}-V_{t-1}\right), \end{array}$$

(46) $$\begin{array}{l} \;\;\;\;\;\;\;\;{ℜ}_{t}^{T}\left(\zeta \right)=\left(1+Rd\right){T^{\prime \prime }}_{t-1}+\frac{1}{2}\mathrm{Pr}\sum_{j=0}^{t-1}\left({U^{\prime }}_{t-1-j}T_{j}\right)-\mathrm{Pr}\sum_{j=0}^{t-1}\left(U_{t-1-j}{T^{\prime }}_{j}\right)-\\ \mathrm{Pr}S\left(2T_{t-1}+\frac{\zeta }{2}{T^{\prime }}_{t-1}\right)+Br\left(1+\frac{1}{\psi }\right)\left(\frac{1}{4}{U^{\prime \prime }}_{t-1}^{2}+{V^{\prime }}_{j}^{2}\right)+MBr\left(\frac{1}{4}{U^{\prime }}_{t-1}^{2}+V_{t-1}^{2}\right), \end{array}$$

(47) $${ℜ}_{t}^{C}\left(\zeta \right)={C^{\prime \prime }}_{t-1}+\frac{1}{2}Sc\sum_{j=0}^{t-1}\left({U^{\prime }}_{t-1-j}C_{j}\right)-S\left(2C_{t-1}+\frac{\zeta }{2}{C^{\prime }}_{t-1}\right)-Sc\sum_{j=0}^{t-1}\left(U_{t-1-j}{C^{\prime }}_{j}\right)-\gamma ScC_{t-1}.$$

When Χ=0 and Χ=1, we can write

(48) $$U\left(\zeta ;0\right)=U_{0}\left(\zeta \right),\;\;\;\;U\left(\zeta ;1\right)=U\left(\zeta \right),$$

(49) $$V\left(\zeta ;0\right)=V_{0}\left(\zeta \right),\;\;\;\;V\left(\zeta ;1\right)=V\left(\zeta \right),$$

(50) $$T\left(\zeta ;0\right)=T_{0}\left(\zeta \right),\;\;\;\;T\left(\zeta ;1\right)=T\left(\zeta \right),$$

(51) $$C\left(\zeta ;0\right)=C_{0}\left(\zeta \right),\;\;\;\;C\left(\zeta ;1\right)=C\left(\zeta \right),$$

By Taylor’s series expansion

(52) $$U\left(\zeta ;Χ\right)=U_{0}\left(\zeta \right)+\sum_{t=1}^{\infty }U_{t}\left(\zeta \right){Χ}^{t},\;\;\;{\left.U_{t}=\frac{1}{t!}\frac{\partial^{t}U\left(\zeta ;Χ\right)}{\partial {Χ}^{t}}\right|}_{Χ=0},$$

(53) $$V\left(\zeta ;Χ\right)=V_{0}\left(\zeta \right)+\sum_{t=1}^{\infty }V_{t}\left(\zeta \right){Χ}^{t},\;\;\;{\left.V_{t}=\frac{1}{t!}\frac{\partial^{t}V\left(\zeta ;Χ\right)}{\partial {Χ}^{t}}\right|}_{Χ=0},$$

(54) $$T\left(\zeta ;Χ\right)=T_{0}\left(\zeta \right)+\sum_{t=1}^{\infty }T_{t}\left(\zeta \right){Χ}^{t},\;\;\;{\left.T_{t}=\frac{1}{t!}\frac{\partial^{t}T\left(\zeta ;Χ\right)}{\partial {Χ}^{t}}\right|}_{Χ=0},$$

(55) $$C\left(\zeta ;Χ\right)=C_{0}\left(\zeta \right)+\sum_{t=1}^{\infty }C_{t}\left(\zeta \right){Χ}^{t},\;\;\;{\left.C_{t}=\frac{1}{t!}\frac{\partial^{t}C\left(\zeta ;Χ\right)}{\partial {Χ}^{t}}\right|}_{Χ=0},$$

The auxiliary constraints are nominated such that the series (52–55) converge at Χ=1, that is,

(56) $$U\left(\zeta \right)=U_{0}\left(\zeta \right)+\sum_{t=1}^{\infty }U_{t}\left(\zeta \right),$$

(57) $$V\left(\zeta \right)=V_{0}\left(\zeta \right)+\sum_{t=1}^{\infty }V_{t}\left(\zeta \right),$$

(58) $$T\left(\zeta \right)=T_{0}\left(\zeta \right)+\sum_{t=1}^{\infty }T_{t}\left(\zeta \right),$$

(59) $$C\left(\zeta \right)=C_{0}\left(\zeta \right)+\sum_{t=1}^{\infty }C_{t}\left(\zeta \right),$$

where

(60) $$\chi_{t}=\left\{\begin{array}{l} 0,\;\;\;\;\;t\leq 1,\\ 1,\;\;\;\;\;\;t>1. \end{array}\right.$$

### Convergence of HAM

**Figure 2:**
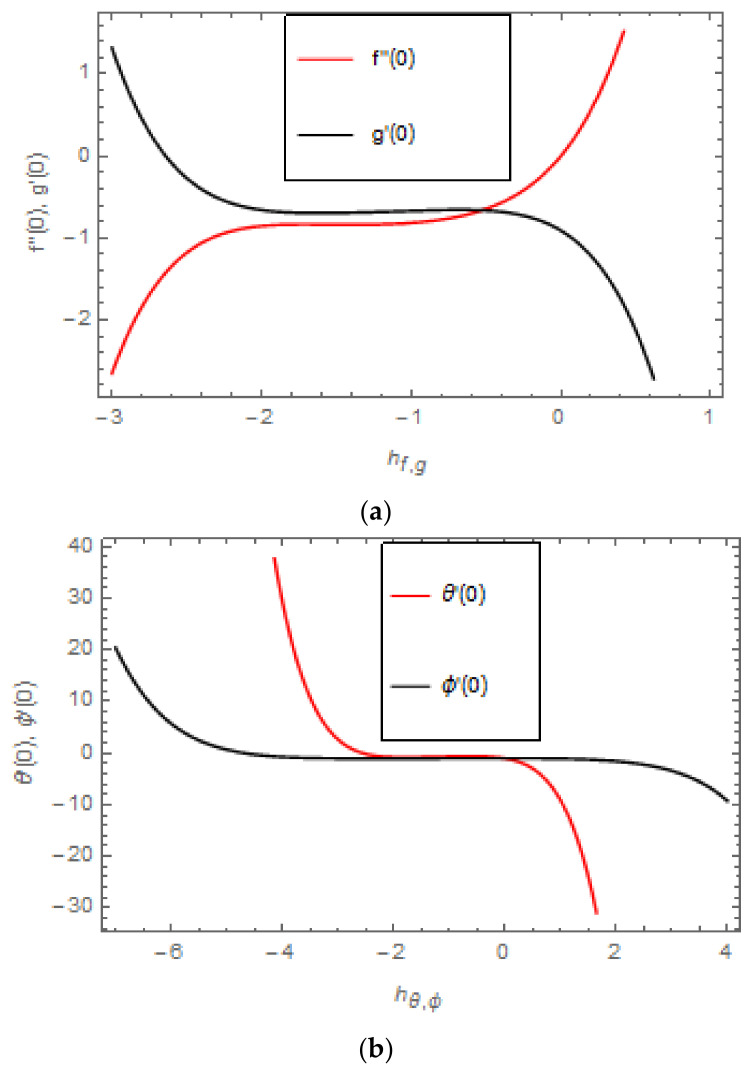
(**a**) ℏ− curves for $f^{\prime \prime }\left(0\right)\;\;\;\mathrm{and}\;\;g^{\prime }\left(0\right)$; (**b**) ℏ− curves for $\theta^{\prime }\left(0\right)\;\;\;\mathrm{and}\;\;\phi^{\prime }\left(0\right)$.

HAM certifies the series solution’s convergence of the demonstrated problem. The assisting factor ℏ acts a dynamic role in regulating and correcting the area of convergence of our series solution. Thus, Figure 2a,b is schemed so as to display the convergence regions for $f^{\prime }\left(\zeta \right)$, $g\left(\zeta \right)$, $\theta \left(\zeta \right)$, and $\phi \left(\zeta \right)$ distributions. The acceptable convergence regions of the supporting parameters $\hbar_{f},\;\;\hbar_{g},\;\;\hbar_{\theta }$ and $\hbar_{\phi }$ are $-2.0\leq \hbar_{f}\leq -0.5$, $-2.0\leq \hbar_{g}\leq -0.5$, $-2.0\leq \hbar_{\theta }\leq 0.0$, and $-5.0\leq \hbar_{\phi }\leq 2.0$.

.

## 5. Results and Discussion

The coupled differential equations are treated analytically by means of HAM. Figure 3, Figure 4, Figure 5, Figure 6, Figure 7, Figure 8, Figure 9, Figure 10, Figure 11, Figure 12, Figure 13, Figure 14, Figure 15, Figure 16, Figure 17, Figure 18, Figure 19, Figure 20, Figure 21, Figure 22, Figure 23, Figure 24, Figure 25, Figure 26, Figure 27, Figure 28, Figure 29, Figure 30 and Figure 31 are drawn with the help of computed codes in mathematica 10.0. These figures designate the variation in velocities, temperature, concentration, entropy generation, and Bejan number profiles via different indicated domains of dimensionless parameters like Casson, magnetic, Hall, mixed convection, buoyancy ratio, diffusion, chemical reaction, and radiation, and dimensionless numbers like Brinkman, Prandtl, and Schmidt. Additionally, the variation in fluid flow is studied in both circumstances of slip $\left(\beta \neq 0\right)$ and non-slip $\left(\beta =0\right)$ boundary conditions. 

Comparisons of the present analysis with the previous analysis mentioned in the literature by Chamka et al. [10] are presented in Table 2 and Table 3. The surface drag force along primary and secondary velocities components is shown in Table 2. The temperature gradient is shown in Table 3. Here, by means of present results with previous results, a great agreement is observed, which validates our present work. 

### 5.1. Velocities, Temperature and Concentration Functions 

**Figure 3:**
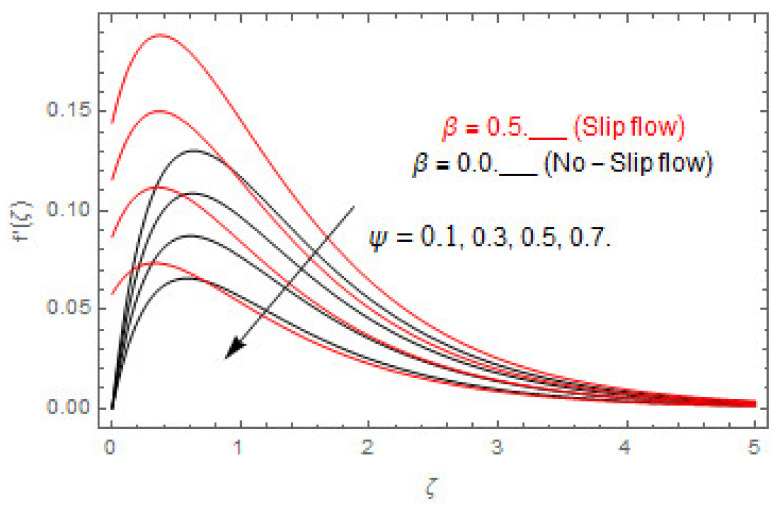
Via ψ.

**Figure 4:**
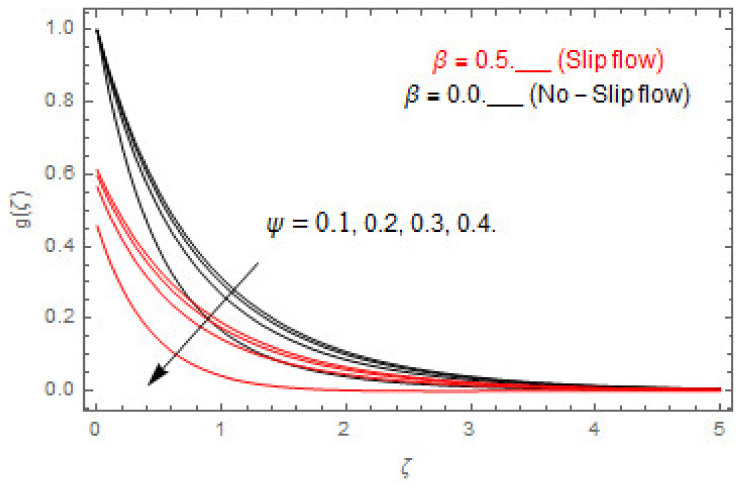
via ψ.

**Figure 5:**
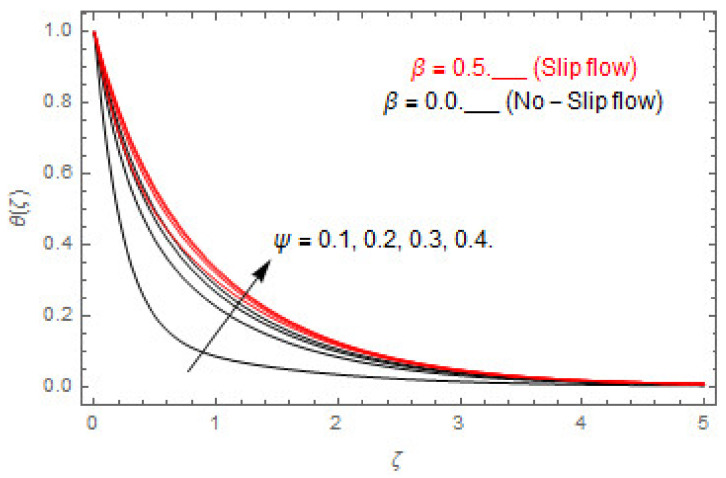
via ψ.

**Figure 6:**
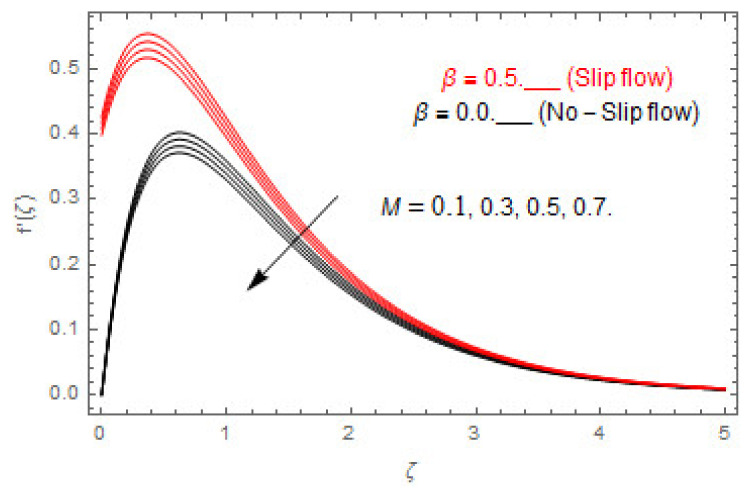
Via M.

**Figure 7:**
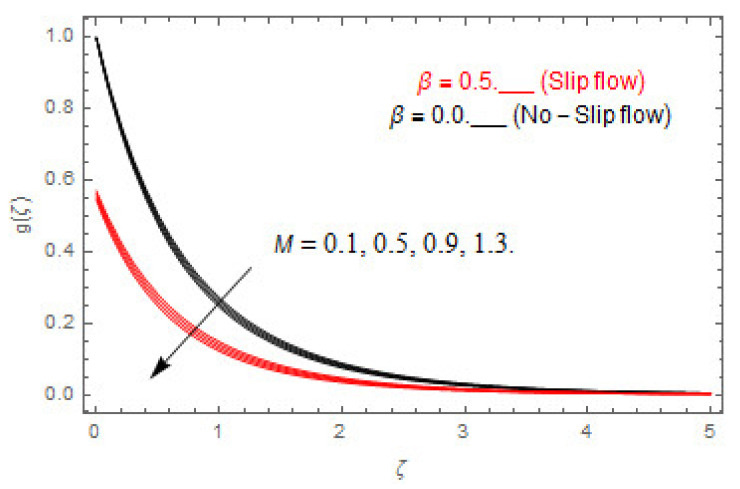
Via M.

**Figure 8:**
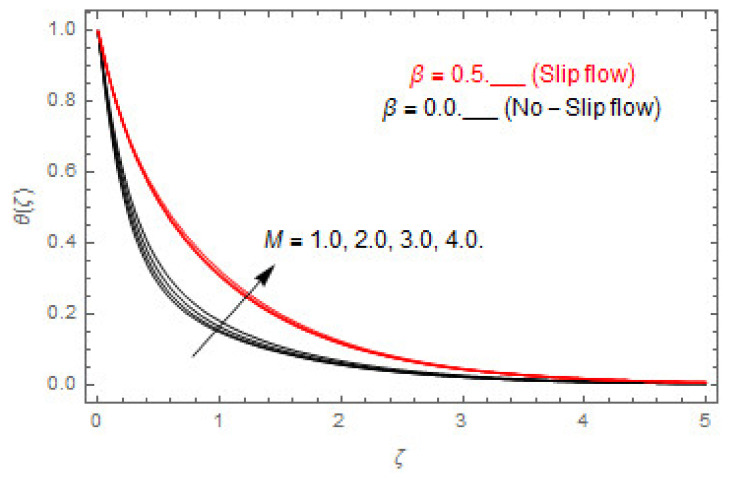
Via M.

**Figure 9:**
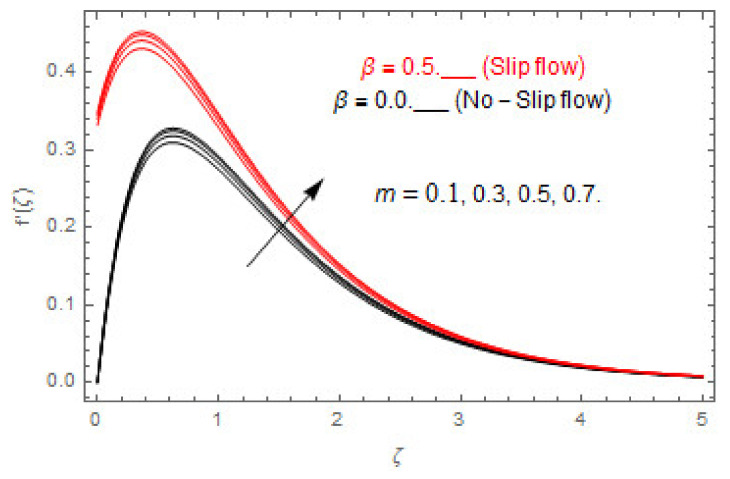
Via m.

**Figure 10:**
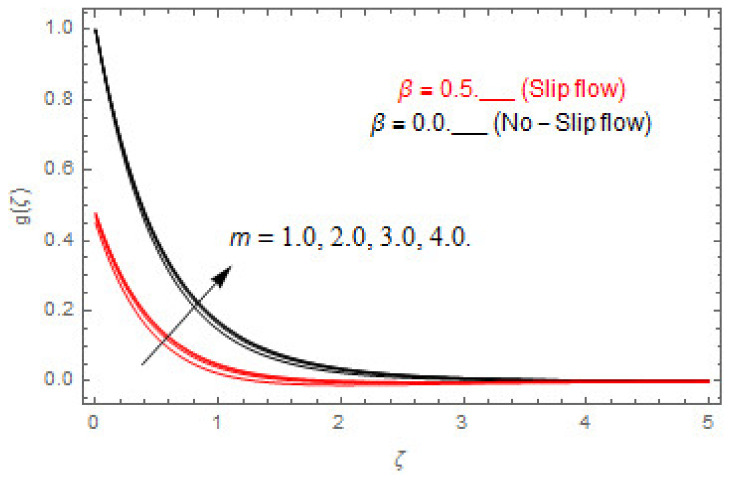
Via m.

**Figure 11:**
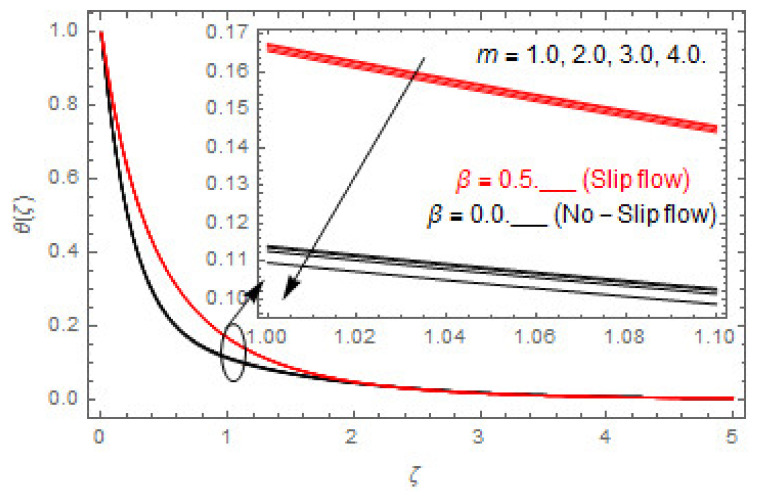
Via m.

**Figure 12:**
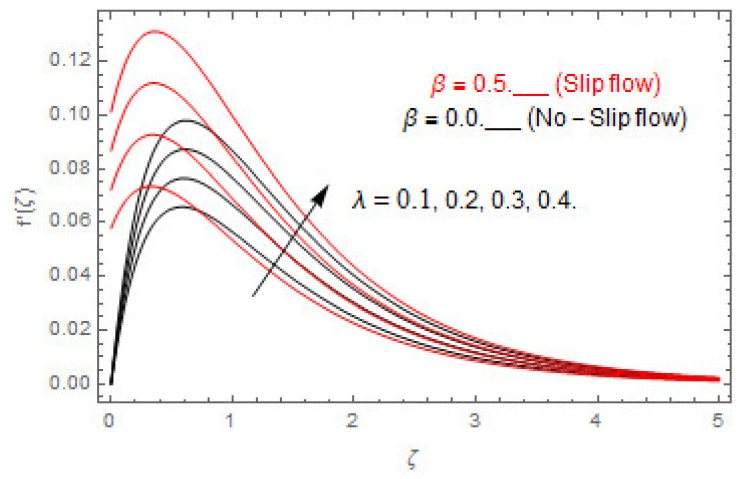
Via λ.

**Figure 13:**
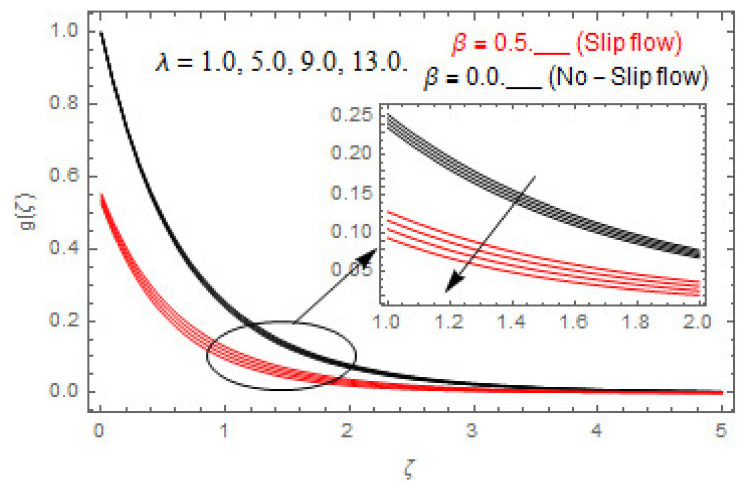
Via λ.

**Figure 14:**
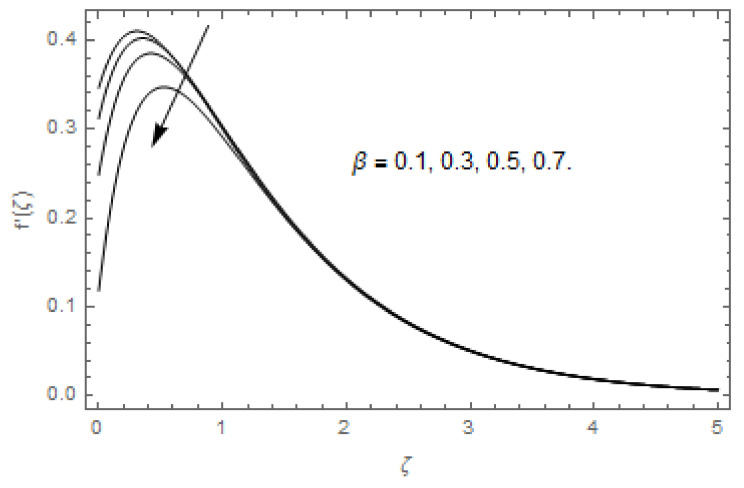
Via β.

**Figure 15:**
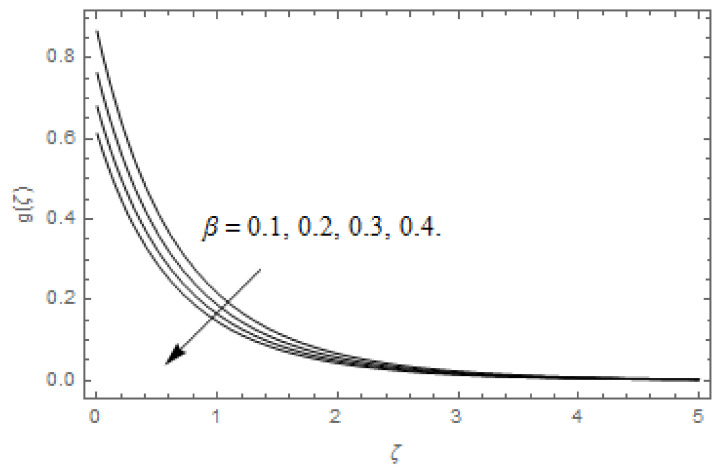
Via β.

**Figure 16:**
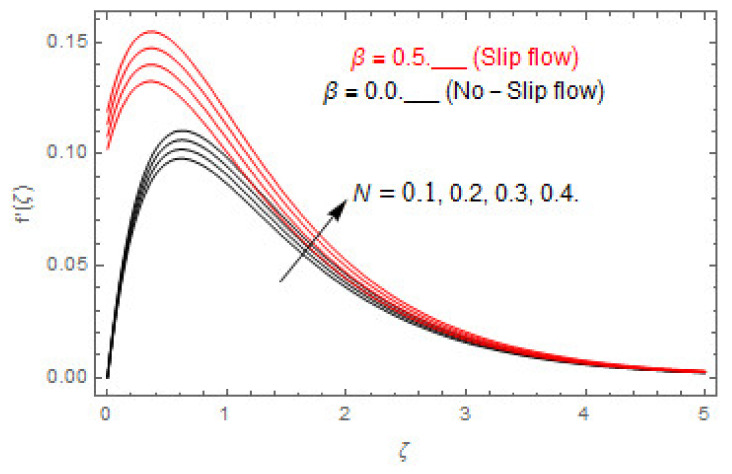
Via N.

**Figure 17:**
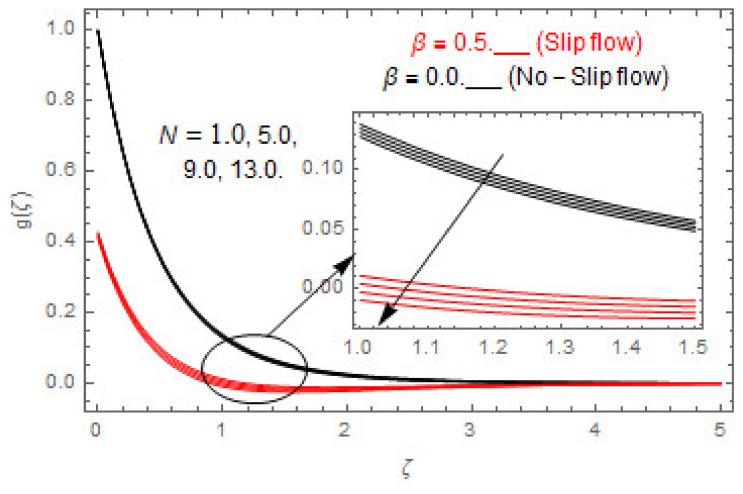
Via N.

**Figure 18:**
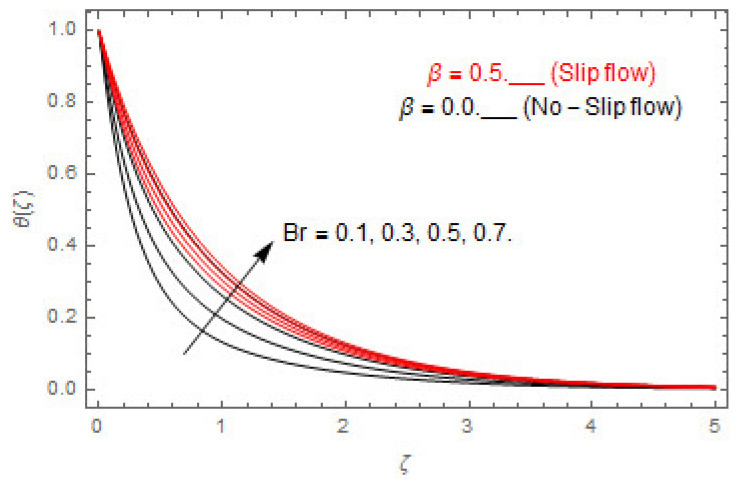
Via Br.

**Figure 19:**
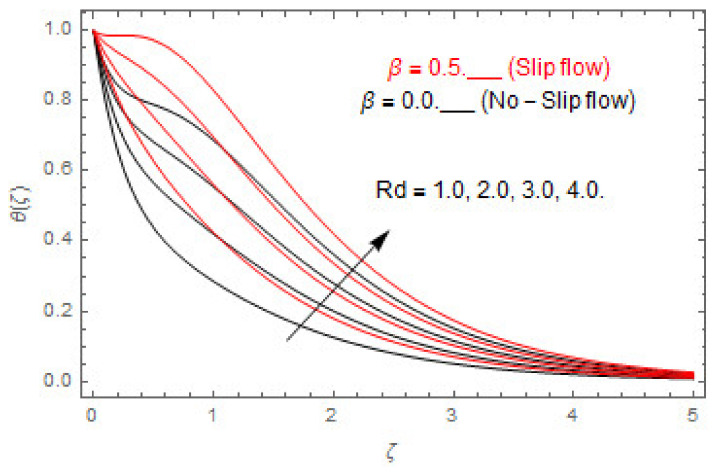
Via Rd.

**Figure 20:**
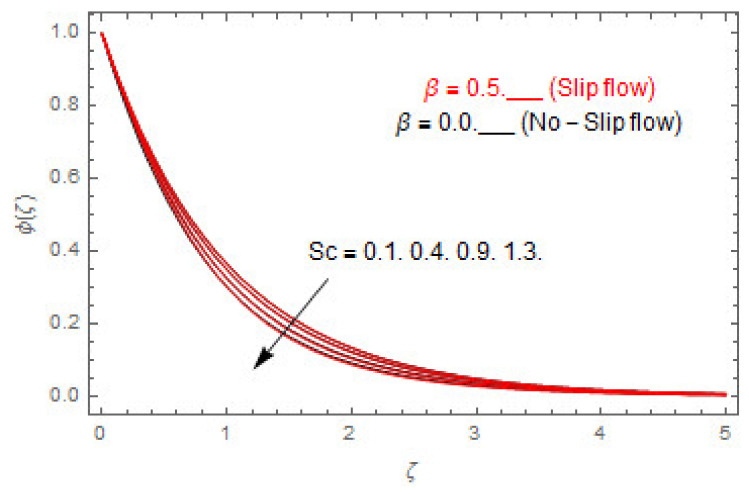
Via Sc.

**Figure 21:**
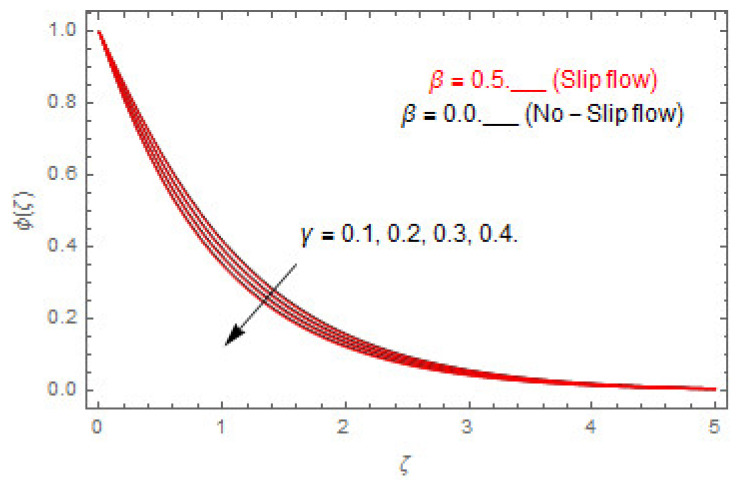
Via γ.

Figure 3, Figure 4, Figure 5, Figure 6, Figure 7, Figure 8, Figure 9, Figure 10, Figure 11, Figure 12, Figure 13, Figure 14, Figure 15, Figure 16, Figure 17, Figure 18, Figure 19, Figure 20 and Figure 21 are displayed to observe the variation in velocities, temperature, and concentration functions via dimensionless parameters. Figure 3, Figure 4 and Figure 5 depict the variation in primary velocity, secondary velocity, and temperature profiles for both (β=0,  β=0.5) cases via Casson parameter ψ. The velocities distributions reduce with higher Casson parameter for both (β=0,  β=0.5) cases. Furthermore, opposite variation in temperature distribution is depicted via Casson parameter. Actually, the plastic dynamic viscosity increases with heightening in Casson parameter, which concludes in the yield stress diminishing. This phenomenon creates an opposing force to the fluid motion and escalates thermal distribution. In addition, it is obvious that the Casson parameter tends to infinity and leads to a Newtonian fluid. Figure 6, Figure 7 and Figure 8 are displayed to depict the variation in velocities and thermal distributions via magnetic parameter. The primary and secondary velocity fields decline with swelling magnetic parameter while a contrary movement is perceived in thermal distribution. Physically, the influence of slip boundary factors and the magnetic factor create the opposing force which in result allow more liquid to pass through the surface and declines the fluid motion in both directions (i.e., $f^{\prime }(\zeta )$ and g(ζ)). This phenomenon is owed to the fact that the Lorentz force creates resistive force to the fluid motion. In addition, the resistive force generates more heat in the fluid flow and therefore the thermal field heightens. The variation in velocities and temperature distributions via Hall parameter are published in Figure 9, Figure 10 and Figure 11. The primary and secondary velocity distributions heighten via Hall parameter, as displayed in Figure 9 and Figure 10. The last terms $\left(\sigma /\left(1+m^{2}\right)\right)$ displayed in Equations (7) and (8) guarantee a decline in conductivity via higher estimations of Hall parameter, which produces damping force that escalates the fluid velocity components. A descending impact of the thermal field via Hall factor is described in Figure 11. It is also depicted that the variation in non-slip flow is more than the slip flow. Figure 12 and Figure 13 determine the variation in primary and secondary velocity distribution via a mixed convection parameter. The primary velocity increases via the mixed convection parameter whereas a contrasting trend is depicted for the secondary velocity of the fluid flow. Greater estimations of the mixed convection parameter escalate the fluid particles’ kinetic energy, and consequently the resistive force to the particles of the fluid deescalates. Therefore, the primary velocity increases, while the secondary velocity decreases. Figure 14 and Figure 15 illustrate the result of slip factor on primary and secondary velocity distributions. Both of the velocities distributions are reduced via higher estimations of slip parameter. This effect is palpable, as we surge the slip factor the velocity distributions of the fluid flow decline. Figure 16 and Figure 17 display the variation in primary and secondary velocity distributions via buoyancy ratio parameter. The primary velocity distribution heightens via higher values of buoyancy ratio parameter, whereas an opposite influence is depicted against secondary velocity distribution. The variation in thermal field via Brinkman number is displayed in Figure 18. Higher estimations of Brinkman number raise the thermal field of the fluid flow. The greater estimations of Brinkman number provide a smaller amount of thermal conduction to the fluid and successively the thermal distribution heightens. Figure 19 portrays the deviation in thermal distribution via radiation parameter. Actually, the greater estimations of radiation factor produce extra heat in the fluid flow and thus the thermal distribution increases. The variation in concentration distribution via Schmidt number is displayed in Figure 20. Actually, the concentration distribution in inversely related with Schmidt number. The intensifying estimations of Schmidt number reduce the thickness of the boundary layer flow. Therefore, the concentration distribution declines. The variation in concentration distribution via chemical reaction factor is presented in Figure 21. Chemical reaction is associated with the rate of mass assignment. The local concentration deescalates with chemical reaction, and therefore the gradient of the concentration and its flux escalates. As a result, the chemical’s concentration reduces with the escalation in chemical reaction parameter.

### 5.2. Entropy Optimization and Bejan Number

**Figure 22:**
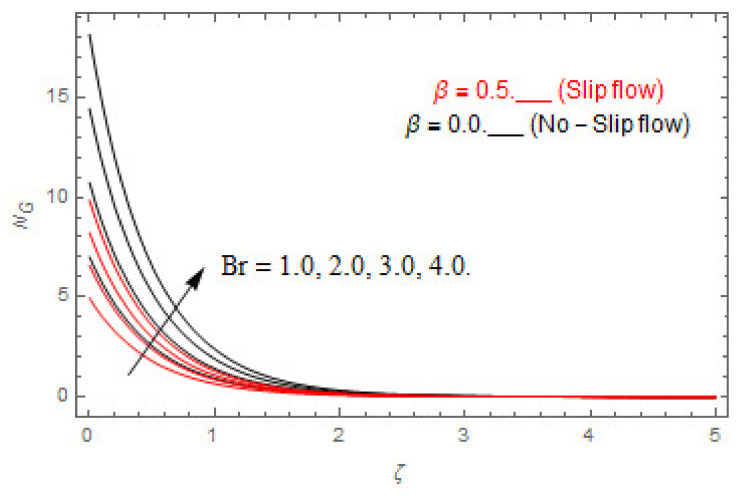
Via Br.

**Figure 23:**
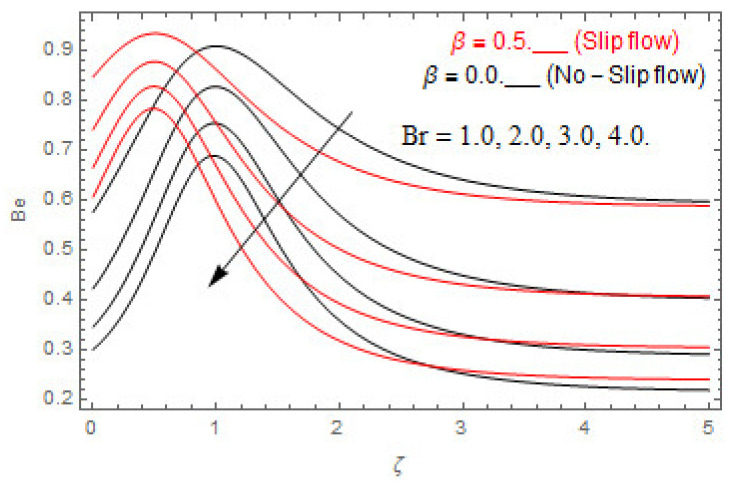
Via Br.

**Figure 24:**
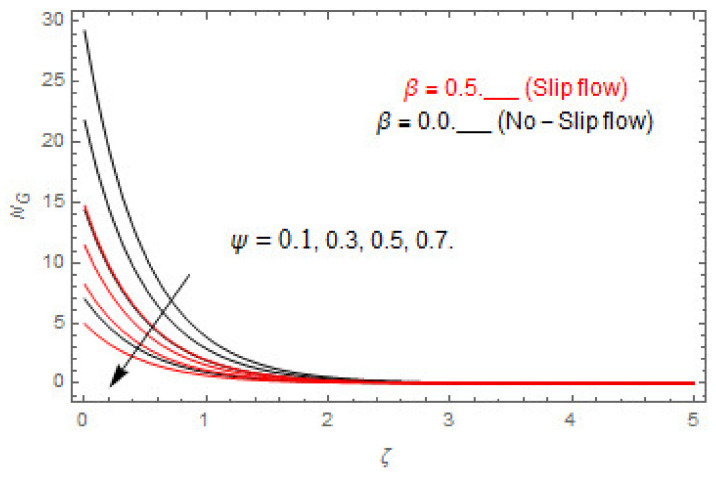
Via ψ.

**Figure 25:**
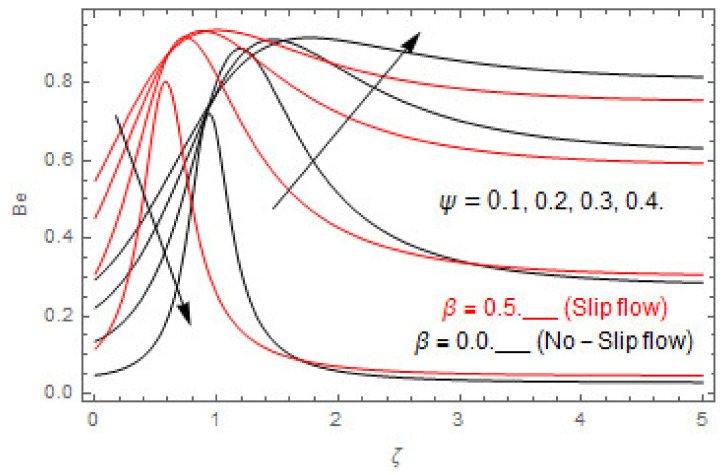
Via ψ.

**Figure 26:**
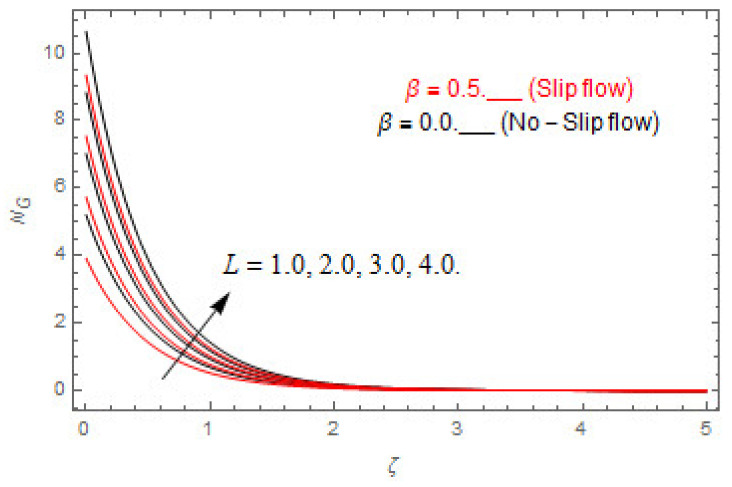
Via L.

**Figure 27:**
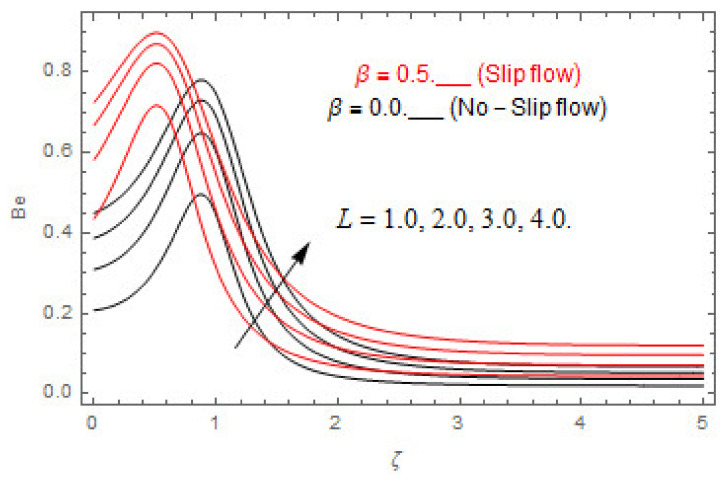
Via L.

**Figure 28:**
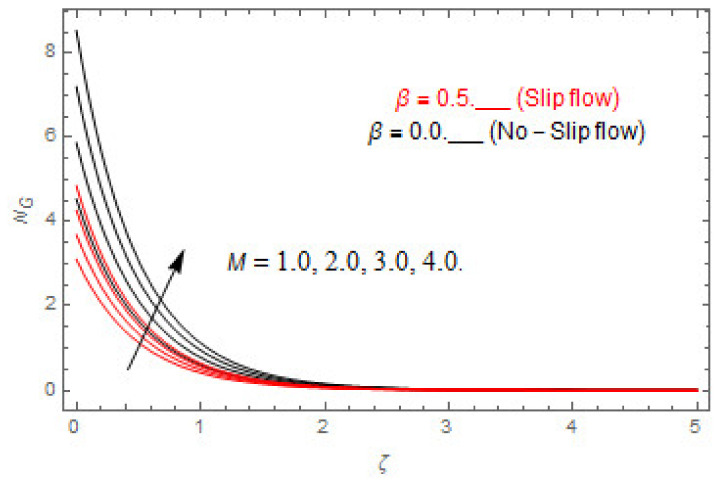
Via M.

**Figure 29:**
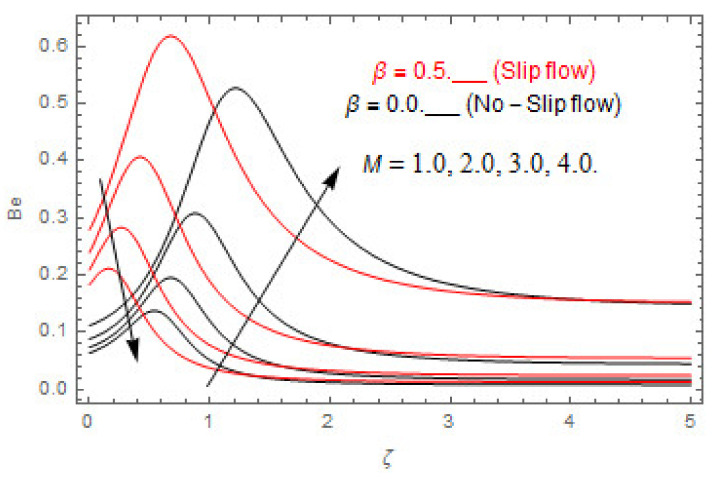
Via M.

**Figure 30:**
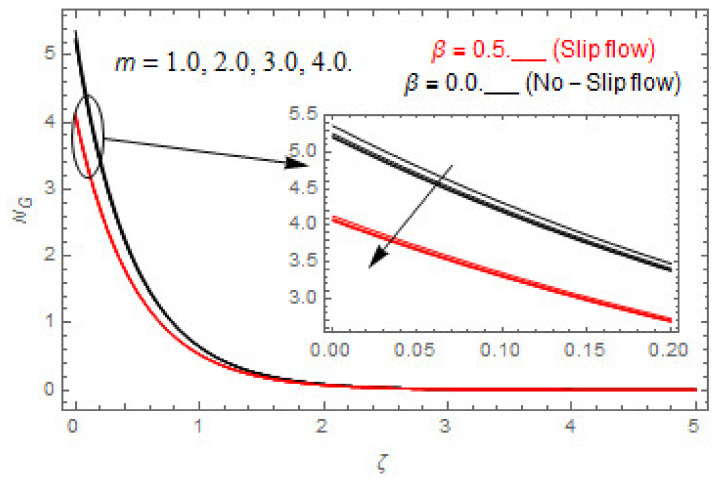
Via m.

**Figure 31:**
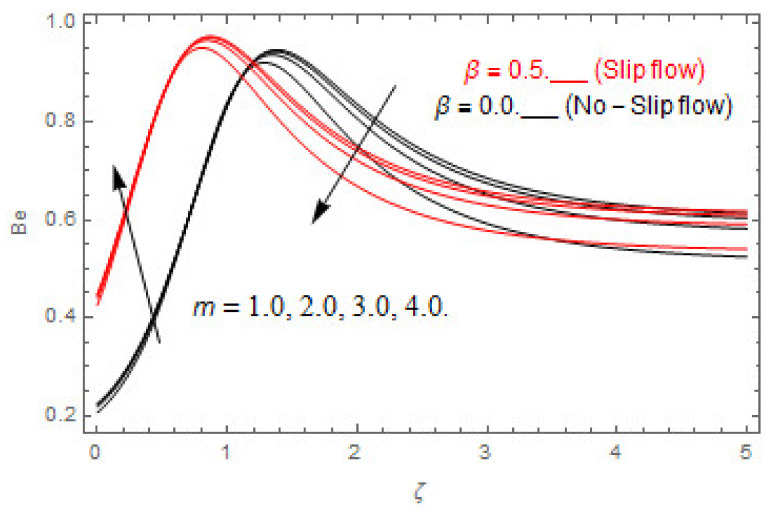
Via m.

The deviations in entropy and Bejan number due to the dimensionless embedded parameters are displayed in Figure 22, Figure 23, Figure 24, Figure 25, Figure 26, Figure 27, Figure 28, Figure 29, Figure 30 and Figure 31. Figure 22 and Figure 23 depict the impact of Brinkman number on entropy and Bejan number, respectively. The higher estimations of Brinkman number escalate the entropy profile, while an opposite effect is observed against Bejan number. Brinkman number engenders heat in the fluid flow moving in the associated region. Thus, the entropy generation increases via Brinkman number. The opposite impact against Bejan number is observed in Figure 23. Figure 24 and Figure 25 are displayed to observe the variation in entropy generation and Bejan number via Casson parameter, respectively. It is obvious that the entropy distribution reduces with greater estimations of Casson factor. Moreover, note that the decline in entropy profile is greater in the case of the non-slip condition compared to the slip condition. A dual impact in Bejan number via Casson parameter is depicted in Figure 25. A decreasing impact is observed via Casson parameter near the surface of the cone while opposite influence is observed after a certain space from the edge of the cone. The variation in entropy profile and Bejan number via diffusion parameter are displayed in Figure 26 and Figure 27, respectively. Both the profiles increase with higher estimations of diffusion factor. Figure 28 and Figure 29 are displayed to observe the variation in entropy distribution and Bejan number via magnetic parameter. Entropy distribution escalates while Bejan number de-escalates at the surface of the cone, and then escalating impact is observed away from the surface of the cone via higher values of magnetic factor. Physically, the increasing magnetic factor yields Lorentz force, which escalates the entropy optimization. The Bejan number drops with higher estimations of magnetic factor near the cone surface while opposite impact is observed after some distance from the cone surface via higher estimations of magnetic factor. Figure 30 and Figure 31 are depicted to see the variation in entropy distribution and Bejan number via greater estimations of Hall factor. It is depicted from the figure that the heightening Hall factor reduces the entropy generation. It is obvious that Hall parameter has a direct influence on the current density and Lorentz force term. Thus, escalating Hall parameter escalates the electrical conductivity of the fluid which in result declines the entropy distribution. The Bejan number escalates near the cone surface where the opposite impact is observed after some distance from the surface of the cone. 

### 5.3. Surface Drag Force, Heat and Mass Transfer Rates

The arithmetical values of skin friction, Nusselt, and Sherwood numbers are illustrated in Table 4, Table 5 and Table 6. As of Table 4, it is depicted that the skin friction along primary direction reduces with magnetic, Hall, buoyancy ratio, and mixed convective parameters, whereas the heightening influence is observed via Casson parameter. The surface drag force along secondary direction decreases via magnetic, Hall, and Casson parameters, whereas it increases via buoyancy ratio and mixed convection parameters. As of Table 5, the Nusselt number de-escalates with higher estimations of magnetic, thermal radiation parameters, and Brinkman number, while it escalates via Casson parameter. As of Table 6, it is perceived that the Sherwood number reduces with higher values of chemical reaction parameter and Schmidt number. 

## 6. Conclusions 

An analysis of unsteady MHD Casson fluid flow with chemical reaction over a rotating cone is presented in this article. Joule heating, thermal radiation, Hall current, and viscous dissipation are considered in this work. Furthermore, the features of entropy generation with first order chemical reaction are examined. The nonlinear system of equations is tackled with HAM. The key points are enumerated underneath.

1. Primary velocity distribution reduces with heightens in Casson, velocity slip, and magnetic factors, whereas the reverse trend is observed against Hall, mixed convection, and buoyancy ratio parameters.
2. Secondary velocity distribution moderates with escalation in Casson, magnetic, mixed convection, velocity slip, and buoyancy ratio parameters, whereas the escalating impact is observed with increasing Hall parameter.
3. Temperature distribution decreases with increasing Hall parameter, whereas the rising impact is observed with escalation in Casson parameter, magnetic parameter, Brinkman number, and thermal radiation.
4. Concentration distribution decreases with increasing Schmidt number and chemical reaction parameter.
5. Entropy generation decreases with increasing Casson parameter and Hall parameter, while it increases with Brinkman number, diffusion parameter, and magnetic parameter.
6. Bejan number decreases with increasing Brinkman number and Hall parameter, whereas the increasing impact is detected with Casson parameter, diffusion parameter, and magnetic parameter.

## Figures and Tables

**Table 1:** Studies on the fluid flows (√ = Effect is present, × = Effect is not present).

Reference #	Fluid Type	Hall Current	Entropy Generation	Chemical Reaction
Ref. [44]	Newtonian fluid	×	×	×
Ref. [45]	Casson fluid	×	×	×
Ref. [46]	Casson fluid	√	√	×
Present study	Casson fluid	√	√	√

**Table 2:** Comparison of $f^{\prime \prime }\left(0\right)$ with previous results.

		Chamka et al. [10]		Current Results	
Pr	λ	$C_{fx}$	$\frac{1}{2}C_{fy}$	$C_{fx}$	$\frac{1}{2}C_{fy}$
0.7	0.0	1.0255	0.6158	1.022543	0.615430
0.7	1.0	2.2015	0.8494	2.201024	0.849312
0.7	10.0	8.5041	1.3995	8.504256	1.399221
10	0.0	1.0256	0.6158	1.025543	0.615831
10	1.0	1.5636	0.6837	1.563001	0.683534
10	10.0	2.0281	0.9840	5.082000	0.984555

**Table 3:** Comparison of $-\theta^{\prime }\left(0\right)$ with previous results.

Pr	λ	Chamka et al. [10]	Current Results
0.7	0.0	0.4299	0.429910
0.7	1.0	0.6120	0.612100
0.7	10.0	1.0097	1.399211
10	0.0	1.4110	1.411101
10	1.0	1.5662	1.566110
10	10.0	2.3580	2.358102

**Table 4:** Numerical results of skin friction along primary and secondary directions.

M	m	N	λ	ψ	CfxRex	12CfyRex
0.0	1.0	0.1	0.1	0.1	−2.52320	2.87840
1.0					−3.29992	2.95818
2.0					−4.07658	3.03796
	0.1				−2.80925	3.18393
	0.2				−3.10214	3.17597
	0.3				−3.36287	3.16328
		0.2			−3.40126	3.16332
		0.4			−3.47804	3.16341
		0.6			−3.55481	3.16349
			0.2		−4.16530	3.16414
			0.3		−4.77579	3.16478
			0.4		−5.38628	3.16543
				0.2	−2.28539	1.09194
				0.3	−1.51112	0.67533
				0.4	−1.16689	0.50498

**Table 5:** Numerical results of local Nusselt number.

M	Rd	Br	ψ	$Nu_{x}$
0.0	0.1	1.0	0.1	2.40758
1.0				2.49147
2.0				2.57265
	0.2			2.82906
	0.3			3.08947
	0.4			3.35391
		1.1		3.52500
		1.3		3.86716
		1.5		4.20933
			0.2	2.98787
			0.3	2.65190
			0.4	2.40617

**Table 6:** Numerical results of Sherwood number.

Sc	γ	$Sh_{x}$
0.1	1.0	1.00840
0.2		0.99847
0.3		0.98854
	1.1	0.98553
	1.2	0.98252
	1.3	0.97951

## Nomenclature

Angular velocity	Ω	Gravitational acceleration	g
Hall parameter	m	Plastic dynamic viscosity	$\mu_{B}$
Yield stress	$S_{y}$	Casson parameter	ψ
Velocity components	u, v, w	Coordinates	x, y, z
Semi-vertical angle	$\alpha^{*}$	Velocity slip parameter	$N_{0}$
Thermal coefficient	$\beta_{t}$	Concentration coefficient	$\beta_{c}$
Temperature	T	Concentration	C
Surface temperature	$T_{w}$	Surface concentration	$C_{w}$
Ambient temperature	$T_{\infty }$	Ambient concentration	$C_{\infty }$
Stefan-Boltzman constant	$\sigma^{*}$	Mass diffusivity	$D_{B}$
Specific heat	$c_{p}$	Chemical reaction parameter	$K_{r}$
Reynolds number	Re	Prandtl number	Pr
Grashof number	Gr	Eckert number	Ec
Schmidt number	Sc	Brinkman	Br
Magnetic parameter	M	mixed convection	λ
buoyancy ratio parameter	N	Slip parameter	β
Thermal radiation parameter	Rd	Dimensionless chemical reaction parameter	γ
Velocity gradients	$C_{fx},\;\;C_{fy}$	Temperature gradient	$Nu_{x}$
shear stresses	$\tau_{xz}$, $\tau_{yz}$	Mass gradient	$Sh_{x}$
Heat flux	$q_{w}$	Mass flux	$h_{w}$
Entropy rate	$N_{G}$	Diffusion parameter	L
Temperature difference	$T_{d}$	Concentration difference	$C_{d}$
Dimensionless parameter	A		
